# Quantitative Analysis of the Phase Transition Mechanism Underpinning the Systemic Self-Assembly of a Mechanopharmaceutical Device

**DOI:** 10.3390/pharmaceutics14010015

**Published:** 2021-12-22

**Authors:** Steven Dunne, Andrew R. Willmer, Rosemary Swanson, Deepak Almeida, Nicole C. Ammerman, Kathleen A. Stringer, Edmund V. Capparelli, Gus R. Rosania

**Affiliations:** 1 Department of Chemistry, University of Michigan, Ann Arbor, MI 48109, USA; scdunne@umich.edu; 2 Department of Pharmaceutical Sciences, College of Pharmacy, University of Michigan, Ann Arbor, MI 48109, USA; awillmer@umich.edu; 3 Johns Hopkins Center for Tuberculosis Research, Johns Hopkins University School of Medicine, Baltimore, MD 21205, USA; swanson.rose@gmail.com (R.S.); dalmeid3@jhmi.edu (D.A.); nicole.ammerman@jhu.edu (N.C.A.); 4 Department of Medical Microbiology and Infectious Diseases, Erasmus MC, University Medical Center Rotterdam, 3015 GD Rotterdam, The Netherlands; 5 Department of Clinical Pharmacy, College of Pharmacy, University of Michigan, Ann Arbor, MI 48109, USA; stringek@med.umich.edu; 6 Department of Pediatrics, Skaggs School of Pharmacy and Pharmaceutical Science, University of California, San Diego, CA 92093, USA; ecapparelli@ucsd.edu

**Keywords:** modeling and simulation, pharmacokinetics, small molecules, drug targeting, drug delivery, tuberculosis

## Abstract

Clofazimine (CFZ) is a poorly soluble, weakly basic, small molecule antibiotic clinically used to treat leprosy and is now in clinical trials as a treatment for multidrug resistant tuberculosis and COVID-19. CFZ exhibits complex, context-dependent pharmacokinetics that are characterized by an increasing half-life in long term treatment regimens. The systemic pharmacokinetics of CFZ have been previously represented by a nonlinear, 2-compartment model incorporating an expanding volume of distribution. This expansion reflects the soluble-to-insoluble phase transition that the drug undergoes as it precipitates out and accumulates within macrophages disseminated throughout the organism. Using mice as a model organism, we studied the mechanistic underpinnings of this increasing half-life and how the systemic pharmacokinetics of CFZ are altered with continued dosing. To this end, *M. tuberculosis* infection status and multiple dosing schemes were studied alongside a parameter sensitivity analysis (PSA) to further understanding of systemic drug distribution. Parameter values governing the sigmoidal expansion function that captures the phase transition were methodically varied, and in turn, the systemic concentrations of the drug were calculated and compared to the experimentally measured concentrations of drug in serum and spleen. The resulting amounts of drug sequestered were dependent on the total mass of CFZ administered and the duration of drug loading. This phenomenon can be captured by altering three different parameters of an expansion function corresponding to key biological determinants responsible for the precipitation and the accumulation of the insoluble drug mass in macrophages. Through this analysis of the context dependent pharmacokinetics of CFZ, a predictive framework for projecting the systemic distribution and self-assembly of precipitated drug complexes as intracellular mechanopharmaceutical devices of this and other drugs exhibiting similarly complex pharmacokinetics can be constructed.

## 1. Introduction

In an accompanying research article [1], a nonlinear, two-compartment model [1] was elaborated to optimally capture the pharmacokinetics of clofazimine (CFZ), a lipophilic, weakly basic antibiotic indicated to treat mycobacterial infections [2,3,4]. Additionally, CFZ is a candidate for treating SARS-Cov-2 infection [5,6,7]. CFZ also acts as an immunomodulatory agent by inhibiting the function of various cellular proteins, including the sodium-potassium ATP-ase [3,8,9]. CFZ exhibits an increased volume of distribution with extended dosing, resulting in a prolonged half-life with respect to total drug administered [10]. Over a 20-week treatment period, experiments using mice as a model organism [11] revealed that drug accumulation was most prominent in the spleen, which harbors large numbers of xenobiotic sequestering macrophages. It can be inferred from previous studies that this increase in half-life corresponds to an increasing volume of distribution that resulted from drug precipitation and massive accumulation in macrophages [12]. To accommodate for the increased drug load, increased numbers of xenobiotic sequestering macrophages and splenomegaly have been observed [10,12,13].

Based on quantitative observations employing a combination of microanalytical techniques together with physicochemical characterization of drug precipitates, CFZ accumulation in the spleen occurs through the formation of intracellular Crystal-Like-Drug-Inclusions (CLDIs), which are stabilized within macrophage lysosomes upon long term oral administration [14]. The mechanism underlying CLDI formation involves the precipitation of drug as a crystalline hydrochloride salt form exhibiting varying amounts of molecular disorder [15] following ion-trapping of the protonated weak base within the acidic lysosomes of macrophages [16]. Microscopically, CLDIs begin to appear in mice after three weeks of continuous oral administration. Accordingly, the mechanism underlying CLDI formation and stabilization within macrophages is the most likely and directly measurable candidate mechanism explaining the abnormal pharmacokinetics of the drug, including its dose-dependent, increasing half-life and its varying volume of distribution [12].

## 2. Materials and Methods

### 2.1. Introduction of Nonlinearity into 2-Compartment PK Model

As reported in the accompanying manuscript, in order to obtain additional insights into the biological mechanism driving CLDI formation and the corresponding changes in the volume of distribution with respect to time of administration, we assessed how the different parameters governing the soluble-to-insoluble phase transition of the drug impacted the accumulation of CFZ in the organism. Elaborating on these findings using a parameter sensitivity analysis, in this manuscript we explored how the manner in which the drug is sequestered and retained within the macrophages of the spleen would impact the concentrations of drug circulating in the blood. For this purpose, we focused on modeling the increase in the drug volume of distribution of the spleen with the three-parameter rational square root (RSR) function [1]:

(1) $$f\left(t\right)=\frac{B_{1}\left(t-B_{2}\right)}{2\sqrt{B_{3}+{\left(t-B_{2}\right)}^{2}}}+\frac{B_{1}}{2}$$

**Figure 1:**
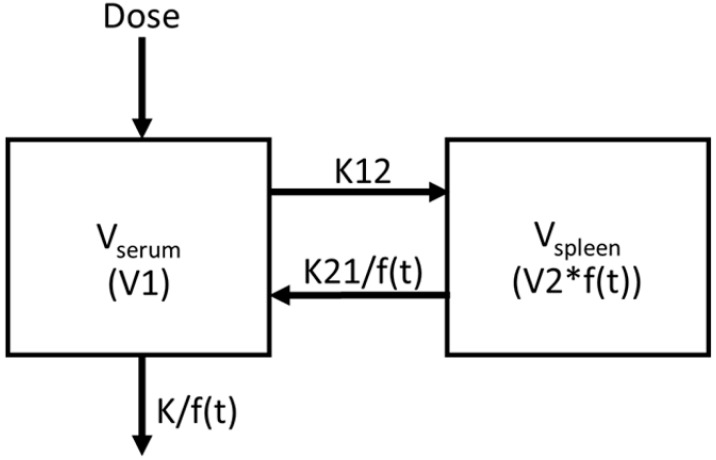
The previously published 2-compartment, context-dependent pharmacokinetic model [1] using the RSR expansion function as f(t).

Equation (1) refers to the expansion function used to apply nonlinearity into the model in Figure 1, and it is also referenced as the ‘RSR function’, ‘expansion function’, or ‘sigmoidal curve’. Incorporating this equation into a 2-compartment pharmacokinetic model (Figure 1), we predicted deviations from the expected results derived from modeling the pharmacokinetics of drug molecules in solution. The results obtained by incorporating this phase transition into the analysis closely paralleled the observed time course of drug accumulation in the serum and spleen. Most conveniently, the RSR function contains three parameters: the first of which captures the upper limit of the function (B_1_) when the spleen’s volume of distribution reaches a maximum; the second, which captures the time of inflection (B_2_) when the rate of CLDI growth changes from increasing to decreasing with respect to time; and a third parameter (B_3_), which affects the overall curvature of the function, reflecting the rate at which drug precipitates out of solution. By varying each parameter individually, the systemic pharmacokinetics of the drug were directly related to the experimentally measured, soluble-to-insoluble phase transition of CFZ in the spleen. Changing B_1_, B_2_, and B_3_ impacted the predicted concentration of CFZ in the serum and spleen, its half-life, and ultimately, the amount of drug that precipitated out and accumulated in the organism each day during the treatment period. By conducting this analysis of CFZ pharmacokinetics under multiple dosing schemes and in both *M. tuberculosis* infected and uninfected mice, these results further our understanding of how the biological mechanisms of CLDI formation, underlying the soluble-to-insoluble phase transition of the drug, ultimately explain the context-dependent pharmacokinetics of the drug in relation to sequestered drug mass and rate of drug administration.

### 2.2. Data Acquisition and Compartmental Pharmacokinetics Modeling

Pharmacokinetics data for the sensitivity analysis were obtained from a previously published 20-week trial with uninfected BALB/c mice that were orally administered 25 mg/kg of CFZ for 5 days each week [11]. Animal procedures were approved by the Animal Ethics Sub-Committee of the University of KwaZulu-Natal (reference numbers 068/13/Animal and 025/14/Animal) [11]. Concentrations of CFZ were measured in the serum and spleen of these mice alongside the masses for each organ. To quantitatively analyze the changes in drug concentration in mice over time, a population pharmacokinetic model was constructed in NONMEM (ver. 7.3.0). Model validation was conducted using bootstrapping in Wings for NONMEM (WFN) to generate parameter estimates, objective function value (OFV), 95% confidence intervals, and coefficients of variation (CV%). After bootstrapping 1000 runs of the RSR function with fixed V_1_ and K_12_, parameter sensitivity was then evaluated. The dose of drug administered and the measured concentrations of drug in serum and spleen were used as input. To illustrate this modeling approach (Figure 1), analysis was focused on serum and spleen compartments. To capture the observed changes in drug mass in the spleen, a three-parameter sigmoidal function f(t) (Equation (1)) was used to model the time-dependent volume of distribution in the spleen, reduced efflux of drug from spleen to serum compartment (K21), and reduced efflux from the system (K_e_). Equation (1) was selected for further evaluation based on lowest Akaike information criteria (AIC) [1] and the direct relationship between the maximal volume of distribution, the inflection point, and the curvature to the corresponding parameters B_1_, B_2_, and B_3_.

### 2.3. Parameter Sensitivity Analysis (PSA)

To analyze how each parameter in the optimized RSR model contributed toward capturing the overall, systemic pharmacokinetics of the drug, parameters B_1_, B_2_, and B_3_, of Equation (1) were varied while holding all other parameters constant. To visualize how the variation in the parameter values affected the pharmacokinetics of the drug, 10-fold increases and 10-fold decreases in the parameters were used to perform simulations using NONMEM. By varying each parameter by an order of magnitude, we accounted for a large variation of possible optimization values. These simulations involved calculating the predicted concentration of CFZ in each compartment over a 20-week time course using the range of different parameter values. In addition, other important measures such as the time-dependent pharmacokinetic variables and volume of distribution in the spleen (V_Spleen_) were recorded. OFV, a -2-log likelihood measure of error between observed and predicted CFZ concentration, was calculated from the simulations. By visual inspection, the results of changing the parameter values were assessed in relation to the curve with the best fit to the concentration vs. time data, which was calculated based on the optimized parameter values and using the same model as previously published [1]. Root mean squared logarithmic error (RMSLE) was used alongside the OFV to further evaluate goodness of fit (Equation (S1)). Confidence intervals, coefficients of variation (CV%), and parameter estimates were generated by bootstrapping analysis.

### 2.4. Total Volume of Distribution and Half-Life Calculations

The total volume of distribution for the two-compartment model was defined as the volume of distribution in compartment 1 (V_serum_) added to the volume of distribution in compartment 2 (V_spleen_), resulting in the equation:

(2) $$V_{\mathrm{total}}=V_{\mathrm{serum}}+V_{\mathrm{spleen}}=V_{1}+V_{2}*f\left(t\right)$$

Half-life was calculated by two independent methods: (1) model predicted half-life, and (2) observed half-life. The model predicted half-life was calculated continuously by adapting the model’s output of elimination rate constant based on a single-compartment, first-order exponential decay model according to the following equation:

(3) $$t_{½}=\frac{\ln\left(2\right)}{K_{e}\left(t\right)}$$

where K_e_(t) corresponds to the variable, time-dependent elimination rate constant calculated by the RSR nonlinear model. The observed half-life was calculated from the terminal rate constants of washout data obtained after loading mice with drug for varying treatment periods and then discontinuing treatment, as performed in the aforementioned study. Serum and spleen CFZ concentration data after the cessation of dosing were measured at 4, 8, 12, 16, and 20 weeks to generate a load-dependent relationship between half-life and duration of dosing. The terminal rate constant from single dose data was used alongside multi-dose data to provide an initial estimate of half-life. Log-linear regression from drug washout data was used to identify the terminal rate constant at the cessation of dosing, which was then converted into half-life. The half-lives with respect to total drug administered were compiled and compared to the predicted half-life using the phase-transition model incorporating the RSR function.

To calculate the total drug mass in the spleen, we used an approximate density of 1 mg/g in the spleen tissue [17] and a calculated average spleen mass of 176 mg from the harvested mice organs.

### 2.5. Dose Dependent Mass Sequestration Analysis

To analyze the mechanistic underpinnings of the phase transition, we utilized additional CFZ pharmacokinetics data in mice under several different dosing regimens [11,12]. Eight different dosing schemes, including three regimens with CFZ loading doses, were used to determine relationships between spleen mass, fractional drug sequestration, and total CFZ drug load. Additionally, *M. tuberculosis* infection status was analyzed as a covariate in the distribution of CFZ. Concentration data for mice infected with aerosolized *M. tuberculosis* H37Rv (ATCC 27294) were obtained from a previously published study [11]. The relationship between dosing regimens was subjected to pharmacometrics analysis utilizing the RSR function, and both linear and log-transformed linear regression conducted in RStudio. Additionally, the cumulative fraction of drug sequestered was evaluated under multiple different dosing regimens. Cumulative fraction sequestered was calculated by dividing the measured mass of drug in the spleen by the total administered mass of drug since the start of dosing.

## 3. Results

### 3.1. Comparative Analysis of Sigmoidal Functions and Utility of the RSR Equation to Describe the Mechanistic Underpinnings of CFZ Pharmacokinetics

In the accompanying manuscript, three different sigmoidal expansion functions f(t) were used to capture the time-dependent change in the volume of distribution. Based on these three different functions, the volume of distribution of the spleen first grows rapidly, then hits an inflection point, and ultimately approaches an upper limit. Consistent with obvious differences in how the shape of the curve is governed by these equations and the corresponding parameters, the parameterization of each of these functions caused notable differences in model output. For the purpose of the mechanistically-relevant, parameter sensitivity analysis performed in this study, the RSR equation (Equation (1)) was used as an expansion function. The RSR equation is parameterized in such a way that the relationships between model parameters and the mechanisms of volume of distribution expansion (and the corresponding drug mass that accumulated in the spleen) was quite simple and direct. Thus, we decided to use the RSR function to obtain additional insights into the relationship between CFZ’s soluble-to-insoluble phase transition and the resulting changes in the systemic concentration of the drug.

Unlike the Exponential Hill Equation and the Logistic Growth Equation, the maximum value achieved by the RSR function corresponds only to B_1_, such that the maximal volume of distribution is directly relatable to B_1_. Furthermore, the RSR equation reaches half its maximal value at time (t*), which corresponds to the time of inflection, where t* = B_2_. Finally, one can estimate the curvature using the maximum slope (f′_max_) where

(4) $${f^{\prime }}_{\max}=\frac{B_{1}}{2\sqrt{B_{3}}}$$

Accordingly, the curvature change is inversely proportionally to the square root of B_3_ and there exists a linear relationship between maximum slope and B_1_.

Notice that in the RSR model, the maximum achieved value and time to reach 50% capacity both correspond to a single parameter (B_1_ and B_2_, respectively). Further, B_3_ can be used to approximate the intensity of curvature for the sigmoid curve, with an increase in B_3_ flattening out the curve. In contrast to the RSR function, the maximum value of the exponential Hill equation is exponentially dependent on E_max_, and t* is dependent on all three parameters. Thus, with the Hill equation, fitting both the derivative and its maximum value is complicated by their mutual dependency on all three parameters, implying single parameter adjustments are not directly correlated to a single pharmacokinetic phenomenon. In the Logistic Growth equation, t* and the maximum slope are both dependent on two parameters. Thus, the Hill and logistic growth equations are more complicated due to the highly ‘coupled’ parameter values, which jointly altered the curvature of the expansion function. The logistic growth model is much less coupled than the Hill model, but it relies on two parameters to determine the time to reach the half capacity inflection point. Thus, as the most “uncoupled” modeling framework, the RSR function greatly facilitated mechanistic interpretation of the quantitative analysis.

### 3.2. Preliminary Analysis of Parameters on Growth Function f(t)

**Figure 2:**
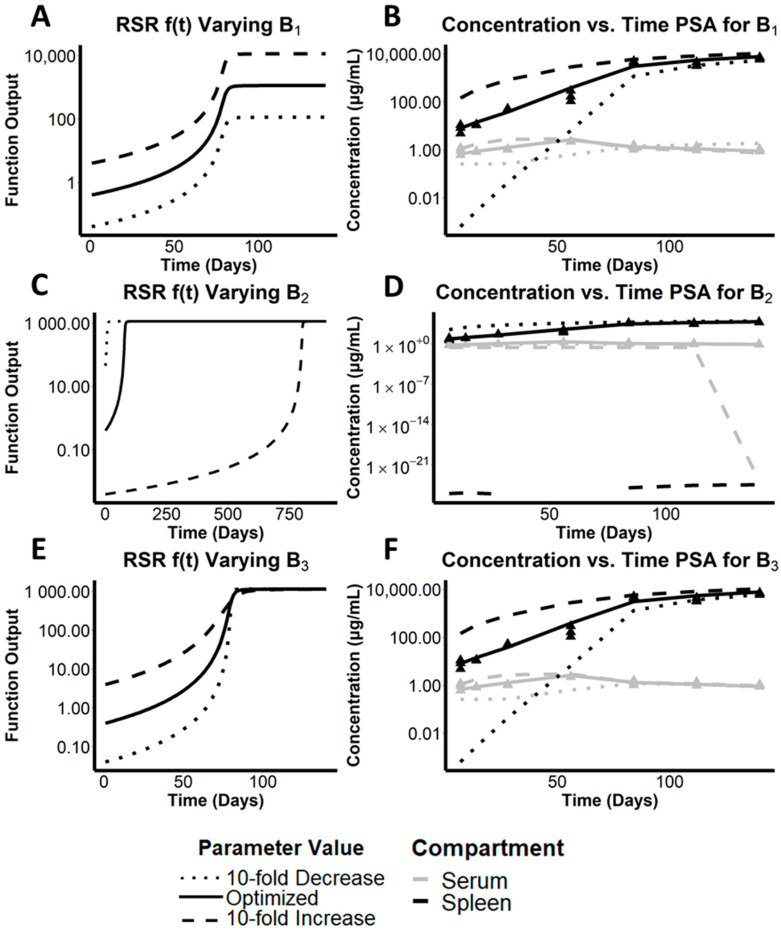
Mathematical analysis of parameters governing the phase transition. Plots (**A**,**C**,**E**) demonstrate the parameterization of the RSR f(t) function by increasing/decreasing each parameter from the optimal values by an order of magnitude, where the solid line is the optimized value, the dotted line is a 10-fold decrease, and the dashed line is a 10-fold increase; (**A**) B_1_ with a 10-fold increase and decrease, (**C**) B_2_ with a 10-fold increase and decrease, and (**E**) B_3_ with a 10-fold increase and decrease. Plots (**B**,**D**,**F**) show the predicted concentration/time changes with respect to a 10-fold increase (dashed lines) and decrease (dotted lines) of parameter: (**B**) B_1_, (**D**) B_2_, and (**F**) B_3_ compared to the predicted concentration over time for the optimized parameters. The triangle points represent the observed data points in both the serum (grey) and spleen (black).

To begin addressing the extent to which different parameters of the RSR equation impacted the shape of the curve, the three parameter values B_1_, B_2_, and B_3_ were varied individually and their effect on the function’s output was plotted and analyzed by visual inspection (Figure 2). Accordingly, B_1_ sets an upper limit for the curve, creating a horizontal asymptote that is approached with continued dosing. As such, B_1_ proportionally increases the magnitude of the RSR function (Figure 2A). In contrast, B_2_ creates a shift in the inflection point of the sigmoidal curve along the x-axis (Figure 2C). Particularly, it achieved this by shifting the time of inflection that corresponded to the time of half-maximal output value (t*), or where the second derivative f″(t) was equal to zero. B_3_ is also related to the expansion time by describing the rate at which the expansion function increases over time (Figure 2E). Nevertheless, large changes must be made to parameter B_3_ to influence the curvature of the function. A decrease in B_3_ corresponds to an increased slope or sharpened curvature while an increase in B_3_ accordingly diminishes slope and soften curvature. Accordingly, there are differences in the sensitivity of the model for each of these parameters due to different scalar changes necessary to show significant difference in curve shape or function value.

### 3.3. Assessing the Effect of the Maximal Cargo Capacity of the Spleen on the Systemic Pharmacokinetics of CFZ

To capture the change in volume of distribution of the spleen compartment, we must look more closely at B_1_. B_1_ determines the upper limit of V_spleen_, as f(t) approaches B_1_ during the 20-week treatment period. Mechanistically, B_1_ reflects a function of biological steady state at a given dose that incorporates spleen growth, increase in the number of xenobiotic sequestering macrophages, and the maximum cargo capacity of the cells that sequester the accumulated drug without leading to overt toxicity. Accordingly, a 10-fold increase in B_1_ would lead to a corresponding increase in the total drug cargo of macrophages in the spleen (Figure 2A, dashed line) relative to the baseline cargo (Figure 2A, solid line). Whereas a 10-fold decrease in B_1_ would lead to a corresponding decreased cargo load of macrophages in the spleen, implying an expected decrease in maximal drug concentration in the organ (Figure 2A, dotted line). Further, B_1_ directly scales f(t), which affects the flow rate from the spleen into the serum and elimination out of the serum, as shown in the model diagram in Figure 1.

Accordingly, by varying the optimized B_1_ parameter using the two-compartment pharmacokinetic model incorporating the RSR equation, the results of the simulations indicated how a change in the total cargo capacity of macrophages in the spleen would influence the systemic pharmacokinetics of the drug. Interestingly, noting difference in magnitude using a logarithmic scale for concentration, the most significant impact of varying parameter B_1_ was observed in the spleen drug concentrations during the first 60 days of drug administration (Figure 2B, black lines). The upper (dashed line) and lower (dotted line) bounds of the spleen concentration deviated by an order of magnitude or more from the optimized B_1_ parameter value that yielded the best fit to the measured drug concentrations in spleen. In contrast, the effect of varying parameter B_1_ on the drug concentrations in the serum was far less significant over the entire 20-week time course (Figure 2B, grey lines), indicating that the circulating concentrations of CFZ were largely independent of the maximal value of the RSR function. We infer that the total amount of precipitated drug and the expansion of the volume of distribution, as well as the maximal carrying capacity of the macrophages in the spleen, are largely independent of serum concentrations. As expected, changes in B_1_ were also reflected in the predicted mass of CLDIs in the spleen (Figure 2B). Accordingly, an increased cargo capacity would be associated with more precipitated CFZ in CLDIs, whereas a decreased cargo capacity would be associated with less precipitated CFZ in CLDIs.

### 3.4. Quantitative Analysis of the Relative Importance of Different Parameters to the Simulated Systemic Pharmacokinetics of CFZ

To establish the relative importance of parameters B_1_, B_2_, and B_3_ on the systemic pharmacokinetics of CFZ, we used RMSLE as a measure of error (Table 1). RMSLE accounts more accurately for changes in order of magnitude by using the difference in natural logarithms between the adjusted parameter model and the optimized model at each timepoint instead of the exact difference used in the more standard root mean squared error (RMSE) analysis. RMSLE was highest for an increased B_2_, and second highest from a decrease in B_2_. This indicates that the amount of time that CFZ needs to be dosed to reach the inflection point of the phase transition, and thus the amount of drug exposure that the organism needs to experience the maximal rate of CLDI accumulation, plays the most significant role in terms of its impact on the systemic pharmacokinetic of CFZ.

OFV was also calculated for each parameter sensitivity analysis performed in this study. The OFV is proportional to the sum of the difference in squares between observed concentration data and predicted concentrated data [6], so it functions similarly to standard RMSE, which accounts for direct difference rather than logarithmic or proportional error. It follows that overestimates due to large changes in parameter values could be weighted as higher error than vast underestimates, both of which are expected by varying each parameter by an order of magnitude in the PSA. Accordingly, the highest OFV is observed for an increase in B_1_, and the second highest OFV comes from an increase in B_2_. Thus, changing the upper limit and the time of inflection both become important when accounting for direct differences between the model and observed data. Diagnostic plots and corresponding values for the PSA are shown in Figures S1–S4.

Scaling parameters by changing their order of magnitude through 10-fold increases and decreases suggest that shifts in B_2_ cause the most drastic changes in model output. Conceptually, this parameter controls the highly time-dependent nature of the acceleration of the phase transition event, so it is natural that such a parameter impacts the model so strongly. To further investigate the dependence of the model on B_2_, we generate 95% Confidence Intervals (CIs) for each parameter alongside the coefficients of variation (CV%). Table 1 shows that, when normalized, B_2_ has by far the tightest bound about which we can be 95% confident in the model’s predictive capabilities. This agrees with the earlier hypothesis that the kinetics leading to time of drug precipitation impact the physiological outcomes, such as drug concentration, more than theoretical notions of maximum cargo capacity or rate of precipitate accumulation. This is shown by the much smaller CV% in B_2_ compared to B_1_ and B_3_. Additionally, this sensitivity analysis provides insight into which parameters of expansion functions provide the most robust and replicable estimates.

### 3.5. Estimation of Increasing Half-Life as a Function of Increasing Volume of Distribution

**Figure 3:**
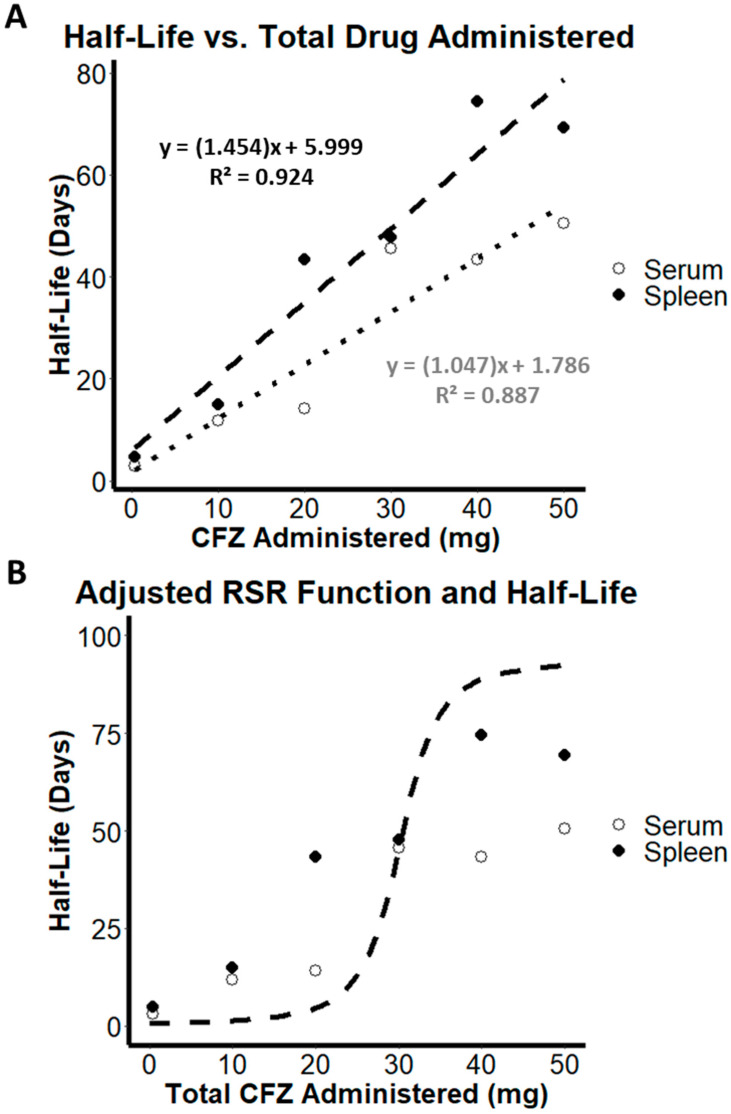
Drug half-life in the organism is related to the cumulative dose administered: (**A**) Load dependent relationship between total CFZ administered and the resulting half-life follows a linear regression in the serum (dotted) and spleen (dashed). (**B**) Calculated serum (open circle) and spleen (closed circle) half-lives plotted against the predicted half-life from the body with the RSR function optimized within 2-fold range of upper and lower observed half-life.

Using washout data from single dose administration and after 4, 12, 16, and 20 weeks of dosing at 25 mg/kg/day [11], log-linear regression was used to estimate the terminal rate constant of the serum and spleen concentrations over time. The half-life of each total CFZ load was thus evaluated and plotted against a linear regression to identify the trend in half-life with increasing drug load (Figure 3A). The half-life of CFZ in the serum increased from 2.9 days after a single dose to 50.6 days after 20 weeks of dosing, resulting in a 17.4-fold increase in half-life in the serum. In comparison, the half-life of CFZ in the spleen increased from 4.9 days after a single dose to 74.5 days after 20 weeks of dosing, resulting in a 15.2-fold increase in half-life in the spleen. While characterized by different rates and extents, the increase in half-life in both the serum and spleen are dependent on the total amount of CFZ administered. By estimating the half-life in both the serum and spleen as a function of total CFZ administered, we can restrict our estimations of the expansion of the volume of distribution throughout the dosing period, to encompass physiological data more accurately. Utilizing the RSR function we optimized the pharmacometrics model with restricted ranges on both the elimination rate constant (K_e_) and the B_1_ expansion variable to result in less than twofold difference from the observed half-life. The restricted RSR function was plotted alongside the calculated half-life values in the spleen and serum (Figure 3B). Under these conditions, the optimized fit of the RSR under-estimated the half-life in low-loading conditions and over-estimated the half-life under higher loading conditions. This indicated that, while the model could strongly predict drug concentration in both the serum and spleen, the overall prediction of half-life from these two compartments can be improved with additional experimental data and the development of a more refined model aimed at better capturing the elimination kinetics of the drug following discontinuation of treatment.

### 3.6. Patterns and Evidence of Load-Dependent Pharmacokinetics from Variable Dosing Data

Based on the insights obtained by focusing on the relationship between the phase transition of CFZ and the systemic pharmacokinetics of the drug, a natural follow up question was: to what extent are these parameters and equations impacted by variation in the dosing regimens? To address this question, eight published CFZ dosing regimens using a range of dosing schemes, including regimens with 2-week loading doses, were analyzed (Table 2) [11,12]. As a result, the total load of clofazimine administered up until each timepoint was evaluated as a covariate rather than the daily dose.

**Figure 4:**
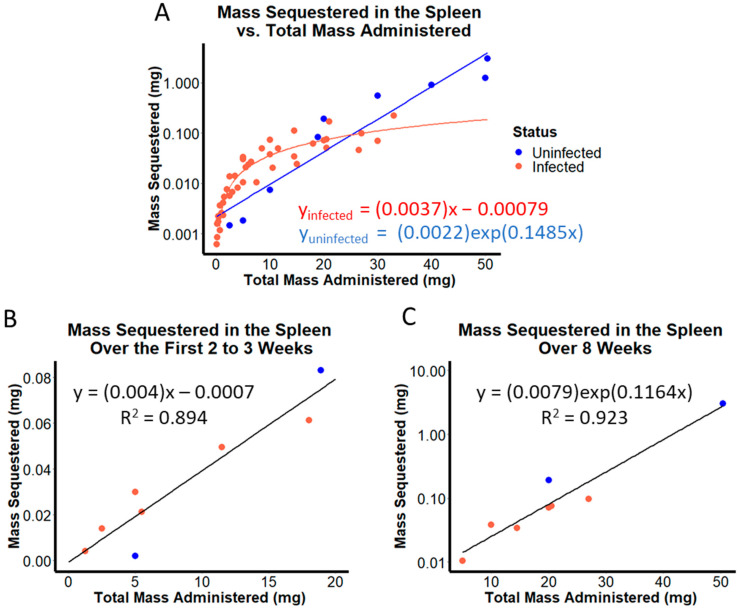
(**A**) Mass sequestered in the spleen as a function of total CFZ administered across 8 dosing regimens with optimization run on the data from uninfected mice (blue) and *M. tuberculosis*-infected mice (red). (**B**) Average mass sequestered after 2 or 3 weeks of drug loading at each of the 8 dosing regimens compared to the total drug administered. (**C**) Average mass sequestered after 8 weeks of drug loading at each of the 8 dosing regimens compared to the total drug administered.

When sequestered drug mass in the spleen was plotted on a logarithmic scale in relation to the total amount of drug administered (Figure 4A), pharmacokinetics of CFZ appeared different in *M. tuberculosis* infected vs. uninfected mice. To better understand these differences in quantitative terms, we performed a nonlinear regression analysis on the plotted data. Accordingly, the uninfected mice showed a log-linear increase in total drug mass sequestered with increasing drug load (blue curve in Figure 4A). In contrast, the infected mice showed a linear increase in total sequestered drug mass with increasing drug load (red curve in Figure 4B). These results suggested that infected and uninfected BALB/c mice accumulated drug at different rates and extents within the observed dosing regimens.

Additionally, when evaluating the mass of drug sequestered in the spleen at 2 and 8 weeks of administration across each of the eight datasets, visual inspection of the plots revealed obvious differences in the dynamics of drug accumulation (Figure 4B,C). Upon further analysis, a moderate linear correlation (R^2^ = 0.89) between CFZ mass accumulated in spleen and total drug administered occurred during the first two weeks of treatment, implying a direct correlation between increasing drug load, and increasing drug mass in the organism. However, when looking beyond 2 weeks of treatment, a strong log-linear relationship (R^2^ = 0.92) between drug mass sequestered and total CFZ administered was observed by the eighth week, implying an exponential increase in CFZ mass sequestered in the spleen with respect to amount of drug administered. The shift from linear to log-linear correlation that occurred as a function of the total amount of drug administered mirrored the results of pharmacokinetics simulations performed with the nonlinear two compartment model incorporating the RSR function, in which nonlinearity from f(t) is much more apt to describe the data obtained at later time points, at which more total drug load has been supplied. These results suggest that in BALB/c mice, infection status, duration of dosing, and total mass administered all influence the fraction of drug sequestered in the spleen at any particular time-point during the dosing period.

**Figure 5:**
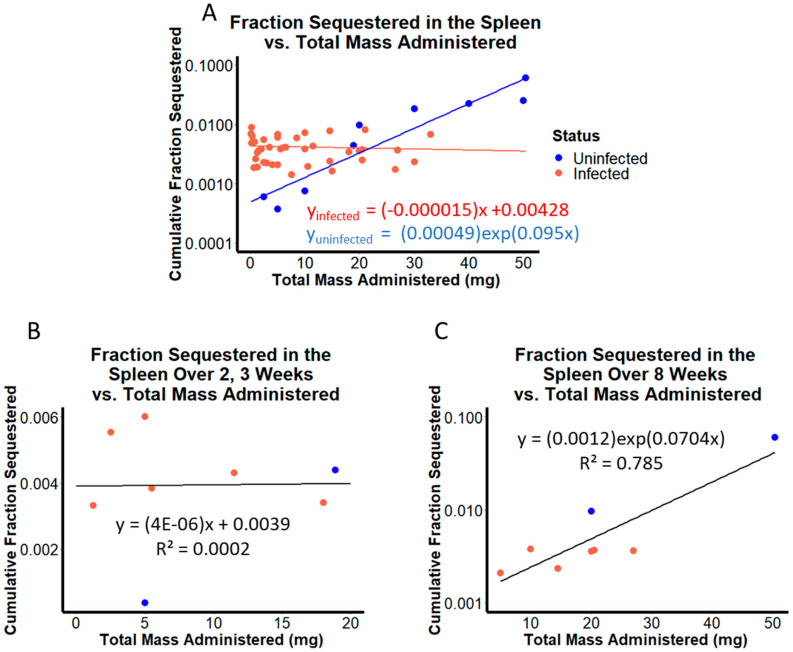
(**A**) Cumulative fraction sequestered in the spleen as a function of total CFZ administered across 8 dosing regimens in *M. tuberculosis* infected (red) and uninfected (blue) BALB/c mice. (**B**) Cumulative fraction sequestered after 2 or 3 weeks of drug loading at each of the 8 dosing regimens compared to the total drug administered. (**C**) Cumulative fraction sequestered after 8 weeks of drug loading at each of the 8 dosing regimens compared to the total drug administered.

Proceeding to examine how the rate of drug sequestration under multiple dosing regimens varies in relation to drug exposure, the cumulative fraction of drug sequestered in the spleen was plotted against the total CFZ load administered (Figure 5). For each of the eight observed dosing regimens, the cumulative fraction of CFZ sequestered in the spleen over the total CFZ administered was predictably different in infected compared to uninfected mice. This relationship was further analyzed by fitting the infected mice data with a linear regression line and uninfected mice data with exponential regression (Figure 5A). The relationship between total drug load and cumulative fraction sequestered after drug administration for 2 and 8 weeks (Figure 5B,C respectively) was then analyzed. After 2 weeks of dosing, there was no relationship between total amount of CFZ administered and the fraction of CFZ sequestered in the spleen over the 2-week dosing period (R^2^ = 0.0002; Figure 5B). However, after 8 weeks of drug loading, there was a log-linear relationship between the total amount of CFZ administered and cumulative fraction sequestered over the 8-week dosing interval (R^2^ = 0.79; Figure 5C). It can be inferred that this observed relationship between cumulative fraction of drug sequestered and total CFZ administered across many different dosing regimens is primarily being driven by CLDI formation.

### 3.7. Investigating the Effect of Infection Status on the Pharmacokinetics of CFZ

**Figure 6:**
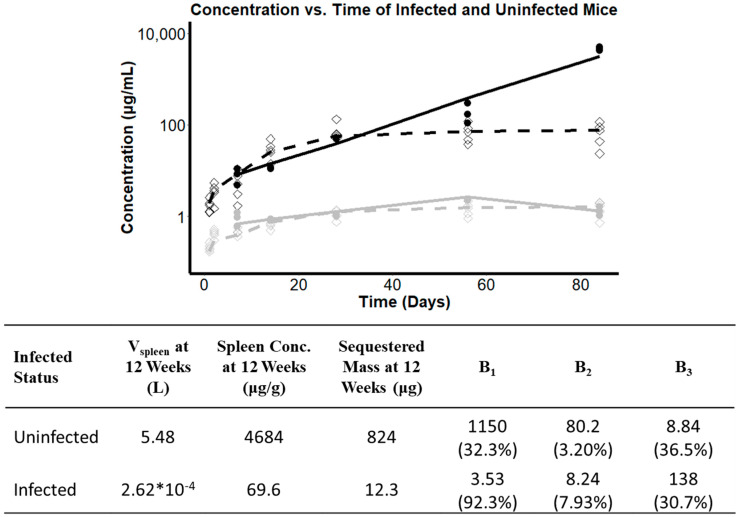
Concentration over time profile in the serum (grey) and spleen (black) over 12 weeks of dosing in mice administered 25 mg/kg/day. Concentration predictions in uninfected mice (solid lines) are shown alongside observed values (solid circles). Concentration predictions in *M. tuberculosis* infected mice (dashed lines) are shown alongside observed values (diamonds). The accompanying table compares the expansion function parameters alongside the relative volume of distribution, spleen concentration, and mass sequestered after 12 weeks of dosing. CV% are expressed in parentheses below the expansion function parameter estimates.

After observing distinct relationships between the total quantity of drug sequestered in the spleen in uninfected and infected mice, additional studies revealed differences in the pharmacokinetics of CFZ in infected and uninfected BALB/c mice undergoing the same dosing regimen of 25 mg/kg/day, 5 days each week for 12 weeks. Comparing the concentration vs. time profiles in infected vs. noninfected mice, the rates and extents of drug distribution were different (Figure 6). This implied that the presence of an *M. tuberculosis* infection changed the pharmacokinetics of CFZ. The reduction in CFZ sequestration that resulted from *M. tuberculosis* infection can be interpreted in terms of a delayed phase transition, which does not expand the cargo capacity of the drug nearly as much as in the uninfected state, causing parameters B_1_ and B_2_ to decrease, and B_3_ to increase, consequently leading to f(t) becoming smaller and increasingly linear.

## 4. Discussion

To summarize, PSA was performed alongside dose dependent analysis of *M. tuberculosis*-infected and uninfected BALB/c mice to study how the context-dependent pharmacokinetics of CFZ vary over a long-term treatment period. By using the RSR equation to model an expanding volume of distribution in a two-compartment model, the pharmacokinetics parameters governing the soluble-to-insoluble phase transition of CFZ in mice spleen could be mechanistically associated with the dose- and time-dependent, systemic pharmacokinetics of this drug. For the most part, the serum concentrations of CFZ were largely determined independently from parameters governing the soluble-to-insoluble phase transition of CFZ and its accumulation as intracellular CLDI precipitates within macrophages. The increase in the half-life of CFZ was expectedly coupled to the expansion of the drug volume of distribution, which can be explained by the thermodynamic and cellular mechanisms responsible for the precipitation of the drug within macrophages [10,12,16]. The modeling approach presented here suggests that the actual concentrations of the drug in the blood are minimally affected by the mechanisms governing the phase transition of the drug throughout the dosing period. To the extent that drug precipitation within macrophages may account for the majority of CFZ accumulation in the organism, there is no reason to expect a steady state of insoluble CFZ is achievable unless the biological, maximal drug cargo capacity of all the macrophages in the organism is saturated. Nevertheless, once the drug precipitates out in the organism, the actual drug concentrations in blood remain nearly constant and insensitive to variations in the dosing regimens, as the circulating drug levels are determined by the thermodynamic equilibrium of drug present in solution with that present in the insoluble precipitates.

### 4.1. Exploring the RSR Function as a Tool to Describe Soluble-to-Insoluble Phase Transitions in a Population Pharmacokinetics Model

The analysis performed herein identified at least two main causes for the differences in the ability of different sigmoidal equations to fit CFZ’s context-dependent pharmacokinetics data and then relate the parameter values to the observed soluble-to-insoluble phase transition phenomenon: the variation in curve shape and differences in the coupling of the parameters in relation to the most important features capturing the curvature of the function. In the case of variation in curve shape, the Hill equation had its maximum rate of change (inflection point) occurring at a time earlier than the time it takes to reach half its capacity, t*. This distinguished the Hill function from the Logistic Growth and RSR functions, in which inflection time and t* were equal. In terms of the coupling of parameters, modeling the pharmacokinetics of CFZ with the RSR equation facilitated a relationship between the spleen and serum concentrations of the drugs to the mechanistic underpinnings of the phase transition. This is due to the simple and direct correspondence between parameter values and the key features of the curve (the maximal value, the time at inflection point, and the slope at the inflection point). The individual parameters governing the shape of a sigmoidal curve may reflect the mechanistic complexities of a drug that undergoes a soluble-to-insoluble phase transition in the organism, and thus can facilitate the design and analysis of future experiments.

Knowing that parameter B_2_, which reflects the cumulative dose of drug that the organism is exposed to before the spleen exhibits its maximal rate of CLDI formation, can so drastically alter output, further studies of mouse models are warranted to analyze the most physiologically important factors that influence this parameter. In the clinical setting, interindividual variability in B_2_ could lead to nonlinear, load-dependent differences in the number of doses needed for drug to precipitate out in the body. Additionally, changes in both B_1_ and B_2_ caused significant variability in predicted half-life and clearance. As such, estimating these parameters could be used to estimate how long CFZ can be expected to remain in the organism, which could be important for the drug’s pharmacological activity, toxicological effects, as well as assessing the potential for drug–drug interactions.

### 4.2. Infection Status as Covariate in CFZ Accumulation

Many possibilities could account for the differences in drug accumulation and the associated volume of distribution of a drug in infected mice, as seen in this analysis. Potentially, infection status could affect the differentiation or function of xenobiotic-sequestering macrophages through differences in the immune signaling and response mechanisms arising from the infection. Localization of infection to the lungs may also influence the location and quantity of macrophages available to sequester drug. An alternate mechanism to be investigated is the effect of tuberculosis infection on the pH of macrophage lysosomes. Previous research demonstrates that CLDIs are destabilized as the pH is increased [17]. It is well-established in the literature that *M. tuberculosis* infection impairs macrophage function by inhibiting the acidification of the lysosome and phagosome, as well inhibiting the fusion of these compartments [18,19]. In turn, an infection could increase lysosomal pH in macrophages beyond levels found in the macrophages of healthy uninfected individuals.

More elaborate analysis of these pharmacokinetic differences between infected and uninfected mice is complicated for several reasons: one, there was sparse data obtained from infected mice that received dosing past 12 weeks or that were administered greater than 30 mg of CFZ. Two, the number and proportions of macrophages may be different in the uninfected and infected mice. Three, there is not enough data to establish how the number and nature of macrophages in the different organs affects the accumulation of CFZ. It is entirely possible that after enough dosing, infected mice may also accumulate drug exponentially in the spleen, but there simply is not enough data available to analyze.

### 4.3. Load-Dependent Drug Sequestration

By analyzing data obtained from mice administered different dosing regimens under varying conditions, CFZ sequestration appeared both dependent on total CFZ load as well as duration of drug loading. A time-variant correlation between drug sequestration in the spleen and total cumulative drug load occurred during a prolonged course of treatment. In addition to the total drug mass that accumulated in the organism, the fraction of dose sequestered across dosing regimens was analyzed to establish a causal relationship behind the total amount of drug administered and disproportional differences in total drug mass sequestered. Under different dosing regimens, no correlation between drug load and fractional sequestration was observed during the first 2 weeks of treatment. Nevertheless, by 8 weeks of drug administration, a log-linear relationship was observed indicating a load dependent increase in fractional sequestration of CFZ in the spleen. The difference between the load-dependent correlations with increasing drug load is consistent with CLDI formation underlying the context-dependent pharmacokinetic profile of CFZ.

In terms of predicting the total amount of CFZ sequestered in the spleen, the results revealed that the rate of dosing and total drug load could play a pivotal role. The mass of CFZ accumulating in the organism as the insoluble phase will likely be dependent on dose and frequency. Since at smaller doses a larger fraction of the drug exists in the soluble phase, metabolic elimination would exert a greater influence on the overall clearance of drug. While there is an apparent relationship between total drug load and the accumulated mass of CFZ in the spleen, this alone does not account for the full extent of CFZ mass present throughout the organism. The drug likely occupies different ratios in the soluble phase, and different clearance pathways depending on the extent of CLDI formation and distribution throughout the different organs of the body. Expectedly, the kinetics of drug precipitation in macrophages and CLDI formation would likely be influenced by both the rate and amount of dosing, together with the treatment period. This leads to interesting biological questions, which can be addressed in future experiments.

When evaluating half-life with the RSR function, the suboptimal fit is most likely due to the simple model used for estimating the elimination half-life. The elimination rate constant is assumed to decrease at the same rate the volume of distribution increases, whereas drug elimination in an organism may be much more complex. The decrease in half-life may not be directly proportional to increase in volume of distribution since multiple elimination pathways may exist in a dynamic physiological environment. Indeed, considering a single elimination pathway for analyzing elimination kinetics is useful but it is likely an oversimplification of the underlying pharmacokinetics of CFZ elimination, following discontinuation of treatment. More detailed analysis of drug elimination with increasing spleen mass and drug sequestration is another avenue worthy of additional, future research.

Despite the fact this study was conducted on mice, crystalline CFZ precipitation has been shown to occur in many organs of human patients after extended treatment [20,21,22,23], including large splenic accumulation. While we may expect different rates of accumulation and clearance in human patient, the phenomena observed throughout this study is expected to be applicable to humans as well. Considerations of the context-dependent, nonlinear pharmacokinetics of clofazimine described here are important for improved efficacy and reduced toxicity in human patients.

## 5. Conclusions

The results of this quantitative analysis imply that a time-varying volume of distribution expansion function f(t) can be useful for obtaining quantitative, mechanistic insights into context-dependent pharmacokinetics. CFZ exhibits both time- and load-dependent adaptations in drug sequestration alongside variable pharmacokinetics with infected status implicating both a biological and pharmacokinetic rationale for the adaptive pharmacokinetic profile. Based on modeling pharmacokinetics as a function of total cumulative dose of drug administered, incorporating a phase transition leads to a bifurcation in the relationship between drug accumulation and total amount of drug administered after a critical drug load is achieved. This is a distinctively nonlinear phenomenon that cannot be accounted for by the alternative, linear multicompartment models [24]. Arguably, while the existing data are insufficient to arrive at definitive conclusions, the observed trends indicate that modeling CFZ pharmacokinetics using a nonlinear two compartment model with an expanding volume of distribution is useful across many different dosing regimens. With additional experiments exploring the dose dependent soluble-to-insoluble phase transitions, a more accurate model could be constructed utilizing dose dependent covariates on the expansion function parameters, to improve upon our understanding of soluble CFZ clearance from insoluble CLDIs. It is important to note the results presented here were evaluated in mice, under CFZ monotherapy. Future research should aim to evaluate CFZ phase transitions not only in mice, but also in both healthy and infected human subjects, under clinically relevant, multi-drug regimens.

## Figures and Tables

**Table 1:** Quantitative measures of model sensitivity including RMSLE and OFV values for 10-fold changes are represented for each of the expansion function parameters. Optimized model-specific parameters (P*), as well as their 95% confidence intervals (CI) are included for each RSR parameter. CV% was calculated by dividing standard deviation by the mean and multiplying by 100%. Complete parameter estimates available in Table S1.

Parameter	RMSLE for 10-Fold Increase	RMSLE for 10-Fold Decrease	OFV for 10-Fold Increase	OFV for 10-Fold Decrease	P*	CI	CV%
B_1_	0.239	0.321	316.419	209.998	1150	[449, 1150]	32.3%
B_2_	0.749	0.297	335.703	326.066	80.2	[76.7, 82]	3.20%
B_3_	0.237	0.314	317.759	211.809	8.84	[4.92, 13.1]	36.5%

**Table 2:** Eight dosing regimens were evaluated from literature. * Evaluated at 3 weeks instead of 2 weeks. † Infected with *M. tuberculosis* (studies 3–8). References: Studies 1, 3–8 [11], Study 2 [12]. BALB/c mice were used in each of the observed dosing regimens.

Study	Dosing Regimen	Total Duration	Route of Administration	CFZ Load at 2 Weeks (mg)	CFZ Load at 8 Weeks (mg)	Total Drug Load (mg)
1	25 mg/kg/d, 5 days per week	20 weeks	Oral Gavage	5	20	50
2	36 mg/kg/d, 7 days per week	8 weeks	Mixed in Feed	18.9 *	50.4	50.4
3 †	6.25 mg/kg/d, 5 days per week	12 weeks	Oral Gavage	1.25	5	7.5
4 †	12.5 mg/kg/d, 5 days per week	12 weeks	Oral Gavage	2.5	10	15
5 †	25 mg/kg/d, 5 days per week	12 weeks	Oral Gavage	5	20	30
6 †	50 mg/kg for 1 day, 25 mg/kg for day 2 to week 2 (5 days per week), 25 mg/kg/d for weeks 2 through 12 (3 days per week)	12 weeks	Oral Gavage	5.5	14.5	20.5
7 †	100 mg/kg for 1 day, 75 mg/kg for day 2, 50 mg/kg for day 3 to week 2 (5 days per week), 25 mg/kg/d for weeks 2 through 12 (3 days per week)	12 weeks	Oral Gavage	11.5	20.5	26.5
8 †	200 mg/kg for 1 day, 100 mg/kg for day 2, 75 mg/kg for day 3 to week 2 (5 days per week), 50 mg/kg/d for weeks 2 through 12 (3 days per week)	12 weeks	Oral Gavage	18	27	33

## Data Availability

Not applicable.

## Supplementary Materials

The following are available online at https://www.mdpi.com/article/10.3390/pharmaceutics14010015/s1, Table S1: Constant Values in the $THETA Record, Figure S1: PSA Diagnostic Plots, Figure S2: Pharmacokinetic Analysis of B_1_, Figure S3: Pharmacokinetic Analysis of B_2_, Figure S4: Pharmacokinetic Analysis of B_3_.
