# Supplementary material for: Quantitative Analysis of the Phase Transition Mechanism Underpinning the Systemic Self-Assembly of a Mechanopharmaceutical Device
Source: Pharmaceutics. 2021 Dec 22;14(1):15. doi: 10.3390/pharmaceutics14010015 (PMC8780429; doi:10.3390/pharmaceutics14010015)
Supplement: Supplementary file 1 [file pharmaceutics-14-00015-s001.zip › pharmaceutics-1442662-supplementary.pdf]

pharmaceutics

# Supplemental Materials: Quantitative Analysis of the Phase Transition Mechanism Underpinning the Systemic Self-Assembly of a Mechanopharmaceutical Device

**Steven Dunne, Andrew R. Willmer, Rosemary Swanson, Deepak Almeida, Nicole C. Ammerman, Kathleen A. Stringer, Edmund V. Capparelli and Gus R. Rosania**

### Statistical Analysis of Population Pharmacokinetics

To establish the relative importance of parameters $B_1$, $B_2$ and $B_3$ on the systemic pharmacokinetics of clofazimine (CFZ), we used the Root Mean Squared Logarithmic Error (RMSLE) as a measure of error (Supplementary Figure 1A).

$$RMSLE = \sqrt{\sum(\log(y_{predicted}) - \log(y_{actual}))^2}$$

**Equation S1.** Root Mean Squared Logarithmic Error equation

RMSLE more accurately resembles error in order of magnitude by using the difference in natural logarithms between the adjusted parameter model and the optimized model at each timepoint instead of the exact difference used in the more standard Root Mean Squared Error (RMSE) analysis.

### THETA Outputs From NONMEM

The optimized model constants defined in the $THETA record were recorded as well as the OFV in the table below.

**Table 1.** Constant Values in the $THETA Record.

| OFV | $K_e$ | ^$K_{12}$ | $K_{21}$ | ^$V_1$ | $V_2$ | $B_1$ | $B_2$ | $B_3$ |
|---|---|---|---|---|---|---|---|---|
| 188.79 | 0.107 (54.1%) | 0.183 | 2.16 (53.4%) | 2.43 | 0.00533 (2.00%) | *1150 (32.3%) | 80.2 (3.20%) | *8.84 (36.5%) |

^Fixed parameter estimates from the soluble phase model.

*Parameters with ETA values.

### Diagnostic Plots of Parameter Sensitivity Analysis

To further monitor the effect of parameter changes on prediction accuracy, the observed CFZ concentration in each compartment was plotted on the y-axis with a corresponding model-predicted concentration for each data point plotted on the x-axis. A fully optimized model would then have the same observed and predicted concentration for each model, corresponding to the linear regression equation y=x, which has a slope of 1. Models optimized with different parameters can thus be compared by calculating a linear regression line for these plots and comparing slope, with values nearer to 1 corresponding to more accurate models. The optimized RSR model (blue in the plots B, C, and D of Supplementary Figure 1) uses a regression line with slope 0.84 and the altered parameter models show lines with a higher slope or lower slope to indicate underestimation or overestimation, respectively. These plots make even more obvious that variations in $B_2$ affects the to a far more significant extent than the other two parameters.

**A**

| Parameter | RMSLE for 10-fold Increase | RMSLE for 10-fold Decrease | OFV for 10-fold Increase | OFV for 10-fold Decrease |
|---|---|---|---|---|
| $B_1$ | 0.239 | 0.321 | 316.419 | 209.998 |
| $B_2$ | 0.749 | 0.297 | 335.703 | 326.066 |
| $B_3$ | 0.237 | 0.314 | 317.759 | 211.809 |

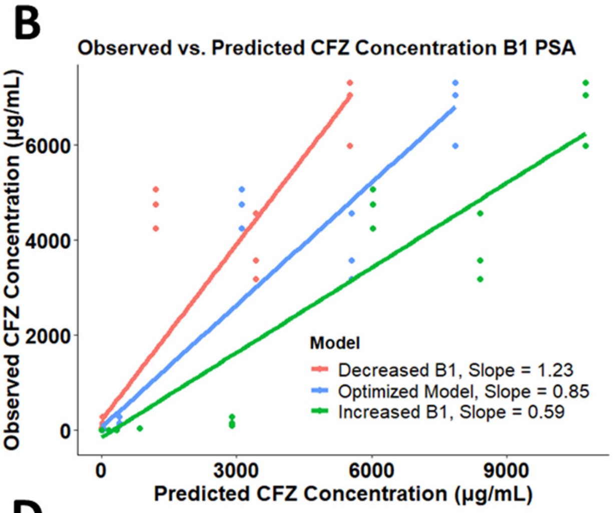


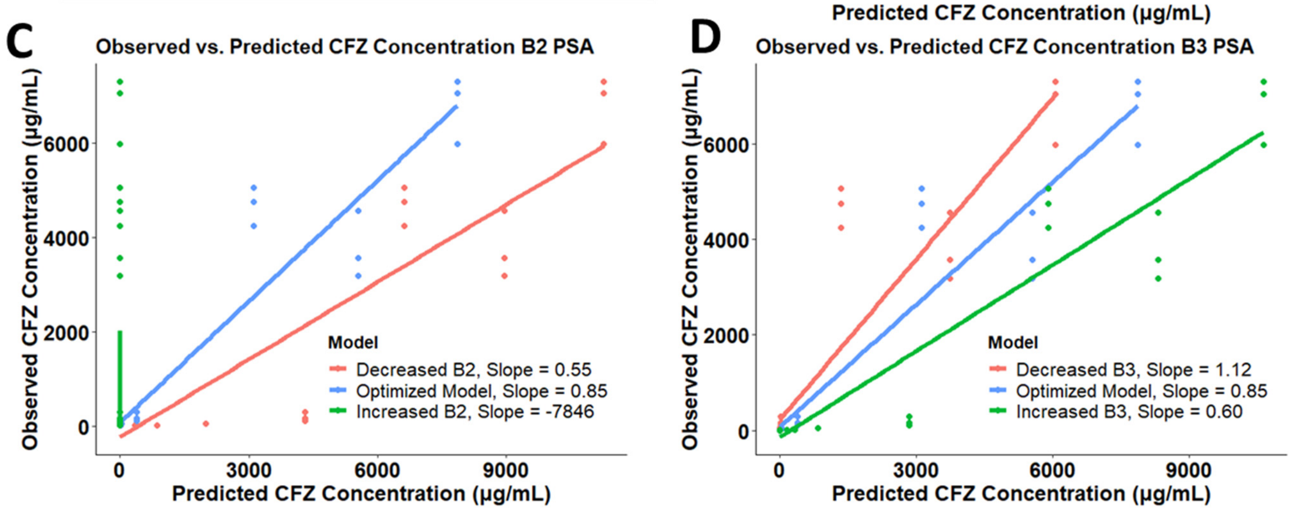


**Figure S1.** PSA Diagnostic Plots. Table A shows the Root Mean Squared Logarithmic Error (RMSLE) for each PSA. B, C, and D show observed CFZ concentration plotted against model-predicted CFZ concentration with regression lines, where more accurate regression lines produce slopes closer to 1. Plot B shows the PSA results for B1, Plot C for B2, and Plot D for B3. Plots were generated for a 10-fold decrease in parameter value (red), the base model (blue), and a 10-fold increase in parameter value (green).

As $B_1$ set a limit for the maximal cargo capacity of the spleen macrophages, a natural question that followed was whether the total volume of the spleen ever approached an upper limit during the 20-week time course of the treatment regimen. Plotting total volume of distribution $V_{total}$ over time, we observed that in the optimized model, the curve begins to flatten around 100 days (Figure 2C, green line). Thus, the $B_1$ parameter is of physiological relevance, and is not simply acting as a scaling factor. Accordingly, splenomegaly and the increase in xenobiotic sequestering macrophage population are candidate biological mechanisms associated with this parameter under the experimental conditions used in this study. Interestingly, the estimated half-life calculated for different values of $B_1$ (Supplementary Figure 2D) all show an upper limit at around approximately 100 days of treatment. While this half-life is an estimate from calculations, it follows that the increased cargo capacity in the spleen will parallel the nonlinear increase in half-life. Indeed, in mice treated with CFZ for many weeks, CLDIs can remain in the mice for several months after treatment has been discontinued [7].

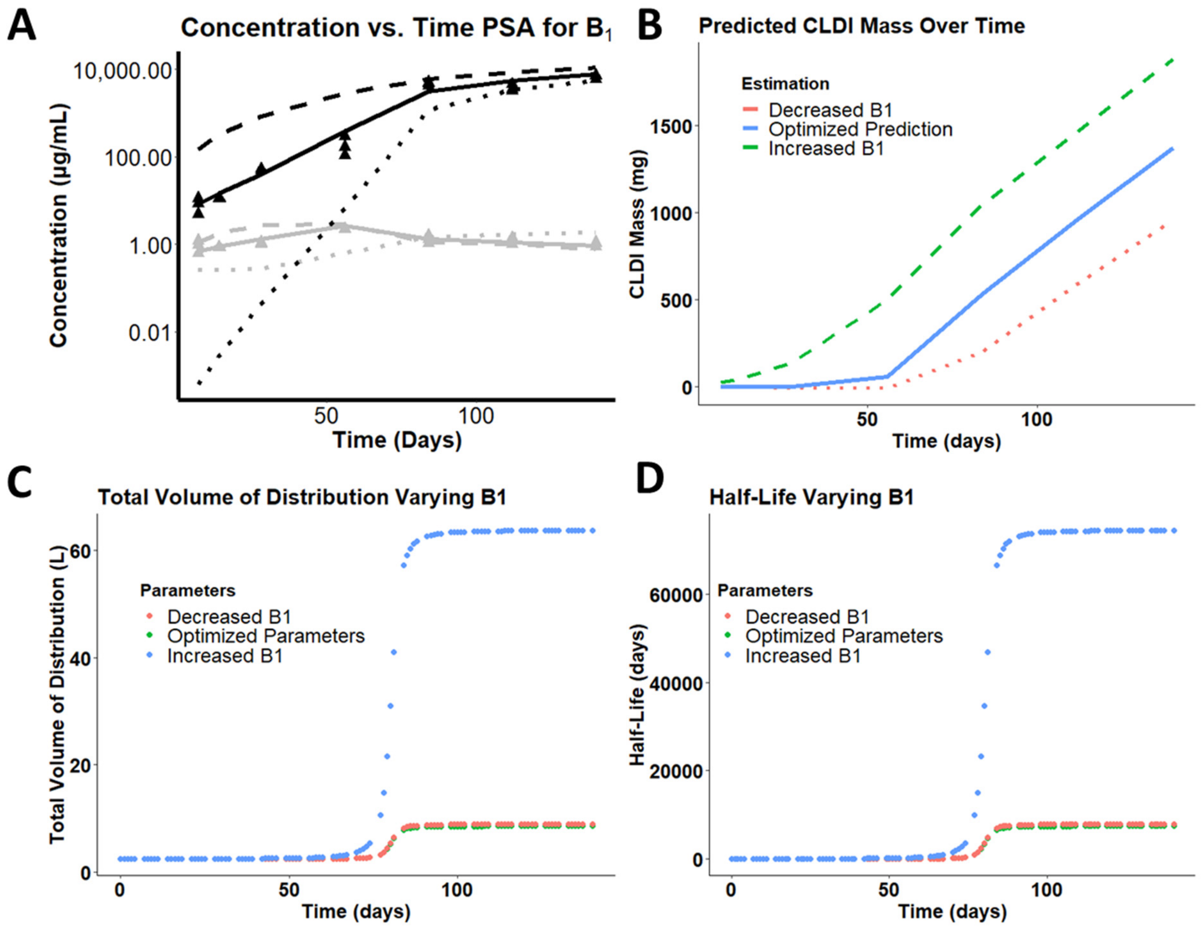


**Figure S2.** Pharmacokinetic Analysis of $B_1$ A) Plot showing the effect of varying $B_1$ on the concentration over time profile of CFZ in the serum (orange) and spleen (blue) based on a 10-fold increase or decrease of $B_1$ relative to the optimally fitted parameter value. B) Plot showing the effect of varying $B_1$ on the predicted CLDI mass in the spleen over time. C) Plot showing the effect of varying $B_1$ on the total volume of distribution. D) Plot showing the effect of varying $B_1$ on the estimated half-life. .

Changing parameter $B_2$ in the RSR function led to a shift in the inflection point of the output of f(t), in which larger values represent a delay in the expansion of the volume of distribution in relation to the amount of time the mice have been treated, as well as the amount of drug administered to the mice. This parameter is also related to how much CFZ must be given to the organism before the spleen and the associated xenobiotic sequestering macrophages that are found therein will begin to accumulate significant amounts of precipitated CFZ as CLDIs. This parameter will also reflect the treatment time at which the maximal spleen cargo loading capacity will be reached. An important pharmacokinetic question regarding $B_2$ is whether the inflection found in f(t) causes real impact on the model. Thus, by changing $B_2$ by an order of magnitude, we can study a range of behaviors in models using with modulated inflection in the f(t) function (Supplementary Figure 3). For example, Supplementary Figure 3C shows the altered $V_{total}$ over time plot with ±10-fold changes in $B_2$, wherein an increased $B_2$ decreased the amount of time that volume of distribution was near its upper limit and a decreased $B_2$ increased the amount of time volume of distribution was near its upper limit. We see that in either of these cases, the inflection curvature has been replaced by more linear behavior. When parameter $B_2$ shifted the curve significantly to later time points, there was very little predicted CFZ mass accumulation in the spleen (Supplementary Figure 3A) with very little associated CLDI formation (Supplementary Figure 3B). While a 10-fold increase in $B_2$ all but eliminated CLDI formation, a 10-fold decrease in $B_2$ created a linear relationship between CLDI

mass and time which overpredicted CLDI formation during early timepoints (Supplementary Figure 3B). The changes induced by increasing or decreasing $B_2$ on the Total volume of distribution (Supplementary Figure 3C) and the half-life (Supplementary Figure 3D) closely paralleled each other, as expected. Accordingly, a faster expansion of the volume of distribution was associated with a faster increase in half-life, and a slower or undetectable expansion in the volume of distribution was associated with no effect on the half-life.

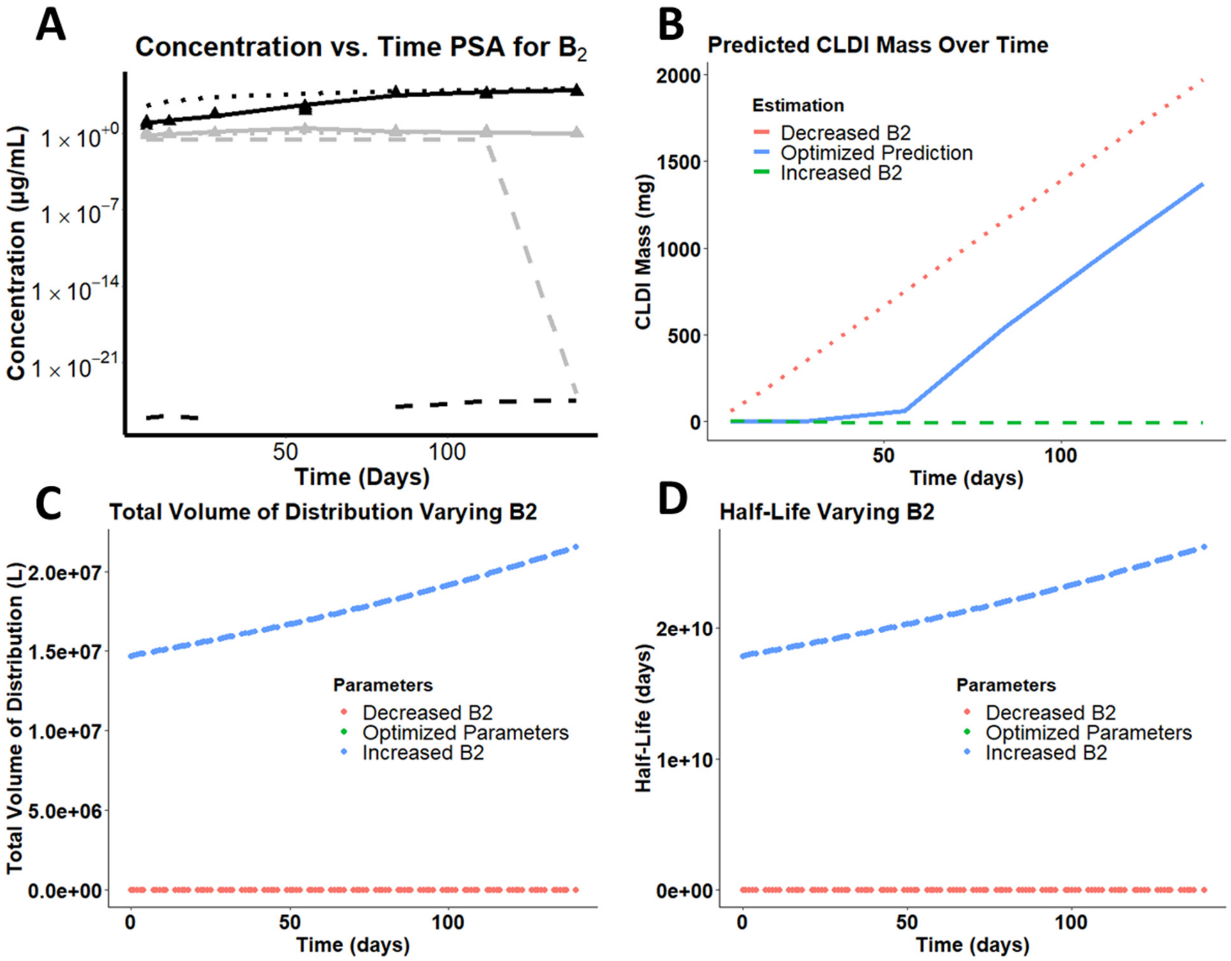


**Figure S3.** Pharmacokinetic Analysis of $B_2$ A) Plot showing the effect of varying $B_2$ on the concentration over time profile off CFZ in the serum (orange) and spleen (blue)based on 10-fold increase (dotted) and decrease (dashed) in the $B_2$ parameter values (relative to the optimally fitted curve). B) Plot showing the effect of varying $B_2$ on the predicted CLDI mass in the spleen over time. C) Plot showing the effect of varying $B_2$ on the total volume of distribution. D) Plot showing the effect of varying $B_2$ on the estimated half-life. .

Parameter $B_3$ in the RSR equation governing function f(t) provided an additional degree of freedom to fully capture the way the phase, affecting the slope and overall curvature of the expansion function. Reflecting the rate of precipitation of CFZ in the spleen as CLDIs, $B_3$ manifests itself in the slope of f(t) at the time of inflection point of the curve generated by f(t). As such, $B_3$ changes the 'maximal phase transition rate,' with an increased B3 decreasing this rate, and thus flattening the shape of the curve governed by function f(t).

To study the effect of $B_3$ on the systemic pharmacokinetics of CFZ, simulations were performed by increasing or decreasing this parameter 10-fold (Supplementary Figure 4). Looking at the effects of $B_3$ on CFZ concentrations in the blood and spleen, most of the impact associated with varying this parameter was on the spleen concentrations of the drug during the first 100 days of treatment (Supplementary Figure 4A). Relative to the

optimized $B_3$ value, increasing $B_3$ mostly affected the amount of CLDI accumulation during the first fifty days of treatment (Figure 4B). The effect of varying $B_3$ on the increase in the Total volume of distribution over time (Supplementary Figure 4C) was relatively less sensitive than the effects of $B_1$ (Supplementary Figure 4C) and $B_2$ (Supplementary Figure 4C). Similarly, the effect on $t_{1/2}$ paralleled the effects on $V_{total}$ (Supplementary Figure 4D) and these effects were small compared to those exerted by parameters $B_1$ (Supplementary Figure 4D) and $B_2$ (Figure 4D).

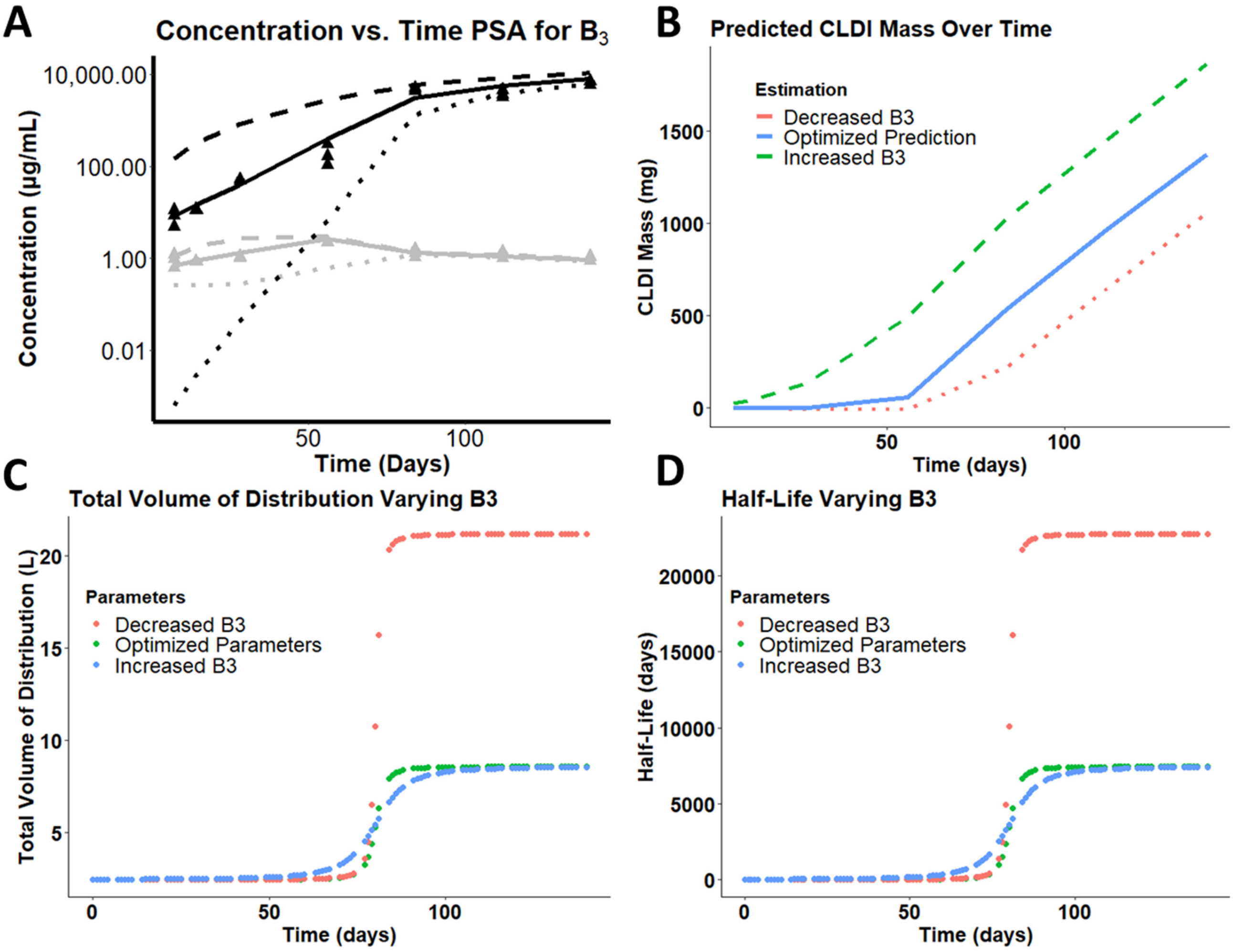


**Figure S4.** Pharmacokinetic Analysis of $B_3$ A) Plot showing the changes in concentration over time profile following a 10-fold increase (red)decrease (green) in $B_3$, using pharmacokinetic data for serum (orange) and spleen (blue). B) Plot showing the effect of varying $B_3$ on the predicted CLDI mass in the spleen over time, C) Plot showing the effect of varying $B_3$ on the total volume of distribution. D)Plot showing the effect of varying $B_3$ on the estimated half-life.
